# Bean and rice meals reduce postprandial glycemic response in adults with type 2 diabetes: a cross-over study

**DOI:** 10.1186/1475-2891-11-23

**Published:** 2012-04-11

**Authors:** Sharon V Thompson, Donna M Winham, Andrea M Hutchins

**Affiliations:** 1 Center for Research on Occupational and Environmental Toxicology, Oregon Health and Science University, 3181 Southwest Sam Jackson Park Road, Portland, OR, 97239, USA; 2 Howell Research, Associates, LLC, P.O. Box 1010, Queen Creek, AZ 85142, USA; 3 Department of Health Sciences, University of Colorado at Colorado Springs, 1420 Austin Bluffs Parkway, Colorado Springs, CO, 80918, USA

**Keywords:** Beans, Type 2 diabetes, Traditional diets, Glycemic response

## Abstract

**Background:**

Around the world, beans and rice are commonly consumed together as a meal. With type 2 diabetes increasing, the effect of this traditional diet pattern on glycemic response has not been studied fully.

**Methods:**

We evaluated the glycemic response of bean and rice traditional meals compared to rice alone in adults with type 2 diabetes. Seventeen men and women with type 2 diabetes controlled by metformin (*n* = 14) or diet/exercise (*n* = 3) aged 35–70 years participated in the randomized 4 × 4 crossover trial. The white long grain rice control, pinto beans/rice, black beans/rice, red kidney beans/rice test meals, matched for 50 grams of available carbohydrate, were consumed at breakfast after a 12 hour fast. Capillary blood glucose concentrations at baseline and at 30 minute intervals up to 180 minutes postprandial were collected. MANOVA for repeated measures established glucose differences between treatments. Paired *t* tests identified differences between bean types and the rice control following a significant MANOVA.

**Results:**

Postprandial net glucose values were significantly lower for the three bean/rice treatments in contrast to the rice control at 90, 120 and 150 minutes. Incremental area under the curve values were significantly lower for the pinto and black bean/rice meals compared to rice alone, but not for kidney beans.

**Conclusions:**

Pinto, dark red kidney and black beans with rice attenuate the glycemic response compared to rice alone. Promotion of traditional foods may provide non-pharmaceutical management of type 2 diabetes and improve dietary adherence with cultural groups.

**Trial registration:**

Clinical Trials number NCT01241253

## Background

*Phaseolus vulgaris* species such as pinto, black and dark red kidney beans with white rice are classic food combinations in many areas of the world, especially in the Caribbean, Latin America, Middle East, and Mediterranean [1]. Epidemiological studies show associations with increased bean consumption and decreased rates/prevalence of chronic diseases including type 2 diabetes [1-3]. In the United States, the Centers for Disease Control estimate that 25.8 million people, or approximately 8% of the population, have type 2 diabetes mellitus [4]. A disproportionate number of Hispanics (11.8%) and African Americans (12.6%) are affected by this disease [4].

Diet and lifestyle changes are the first intervention steps recommended by leading health agencies to prevent and control type 2 diabetes [5,6]. Despite the known benefits of diet and lifestyle change, there is often poor adherence to dietary recommendations [7-10]. In fact, difficulty meeting diabetic dietary guidelines is a frequently reported concern [10], particularly among Hispanic [11-14] and African American type 2 diabetes populations [15,16]. Two adherence barriers often mentioned are exclusion of culturally familiar foods from counseling and diet education materials and the perceived inability to eat the same foods as the rest of the family, e.g. beans and rice [11,12,16].

Beans are known functional foods that are low in fat and high in fiber, vegetable protein, folate, iron, magnesium, zinc, omega-3 fatty acids, and antioxidants [1-3]. They also contain phytate and phenolic compounds that may function in similar ways to α-glucosidase or α-amylase inhibitor type 2 diabetes medications like the oral hypoglycemic agent acarbose [17].

Beans have a low glycemic index (GI) which by definition means they produce a relatively low rise in blood glucose after a meal [17-19]. In contrast, high GI items like long grain white rice can cause postprandial glycemic elevations that are damaging to vascular tissues and other organs [20,21]. Regular white rice consumption has also been linked to an increased risk of type 2 diabetes [22]. Few studies have looked at the acute effects of *P. vulgaris* or common beans on glycemic response as part of traditional meals or in combination with other foods [17,19,20,23].

Since elevated blood glucose is a significant contributor to cardiovascular risk, these findings have important implications for chronic disease risk reduction beyond type 2 diabetes [5,21]. Emphasizing the continued inclusion of culturally familiar beans in the therapeutic diets of persons with type 2 diabetes may decrease postprandial glycemic variability, maintain vascular health, and improve dietary compliance and thus quality of life, especially for immigrants and minorities [9-11,24]. We hypothesized that pinto, black, and dark red kidney beans in combination with long grain white rice would equally reduce postprandial glycemic response in adults with type 2 diabetes.

## Methods

### Study population

Adults aged 35–70 years old with type 2 diabetes managed by metformin or diet/exercise were recruited to participate in the 4 × 4 randomized cross-over trial. Persons using insulin or other diabetic drugs were excluded to minimize potential confounding from multiple hypoglycemic medications with various modes of action. All participants were physician-diagnosed with type 2 diabetes at least 6 months prior to starting the study. The method of diabetes management had to be the same for at least 3 months prior to study entry. Eligible participants had a hemoglobin A1c (HbA1c) value of <10% at screening and had no evidence of condition(s) that would influence their ability to complete the study as determined from medical record analysis. Those with weight changes +/− 5 kg within 6 months, women who were either pregnant or breastfeeding, and individuals with allergy to beans or latex were excluded during the screening process. This study was approved by the University Institutional Review Board, and all participants provided written, informed consent.

**Figure 1:**
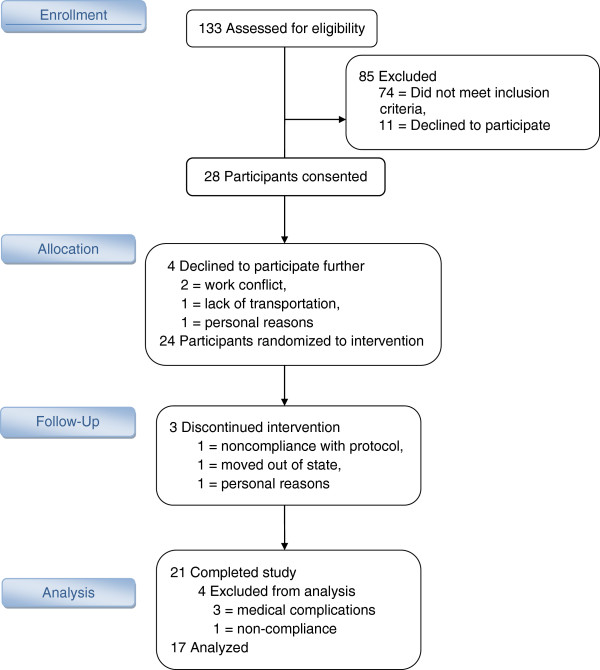
Consort flow diagram.

Twenty-eight individuals with type 2 diabetes enrolled in the study. Twenty-one successfully completed the study in its entirety. Four participants were excluded from final analysis. Three of the latter participants did not fully disclose medical conditions until after they started the study and were ineligible. An additional participant was noncompliant with the pre-test date dietary protocol (See consort diagram, Figure1). Data from 17 individuals (9 men and 8 women) aged 38–70 years were analyzed (Table1). Fourteen of these individuals used the medication metformin to manage their type 2 diabetes, while the other three used dietary methods and/or physical activity.

**Table 1:** Baseline characteristics of study participants (*n* = 17)

**Variable**	**Mean ± SEM**	**Range**
Age (y)	58.6 ± 2.3	(38–70)
Weight (kg)^1^	93.4 ± 4.0	(67.8–120.9)
Height (cm)^1^	170.7 ± 2.1	(154.4–184.5)
BMI (kg/m^2^)^1^	31.9 ± 1.0	(26.9–38.8)
TC (mg/dL)	172.9 ± 7.5	(119.0–220.0)
TG (mg/dL)	156.4 ± 18.8	(68.0–328.0)
HDL-C (mg/dL)	44.4 ± 1.9	(36.0–65.0)
LDL-C (mg/dL)	98.9 ± 5.6	(59.0–133.0)
HbA1c (%)	6.5 ± 0.1	(5.8–7.8)

^1^ Values obtained at study entry. BMI = body mass index, TC = total cholesterol, TG = triglycerides, HDL-C = high density lipoprotein cholesterol, LDL-C = low density lipoprotein cholesterol, HbA1c = hemoglobin A1c.

### Study design

Participants were administered four different test meals separated by one week in this 4x4 randomized crossover study (ClinicalTrials.gov: NCT01241253). At the time of study consent, participants selected a commercial frozen meal, e.g. Lean Cuisine or Marie Callender’s brands. They consumed the same frozen meal for each of the four pre-test evening meals at the same time each evening, in order to reduce any variation in morning glycemic response due to the Second Meal Effect [18,25]. Participants were also given instructions for completing a 24-hour dietary recall for each day before testing and were told to refrain from consuming any alcohol, caffeine or taking part in any physical activity beyond that of their typical daily activities during this time. After consuming the provided meal on the eve of testing, participants drank only water until they arrived at the study location 12 hours later. Upon arrival at the test site, 24-hour dietary recall forms were reviewed by a nutritionist, and participants were confirmed fasting and compliant with study procedures. They were then weighed using a digital scale (Seca Model 880, Hamburg, Germany). Standing height was assessed at the first test day meeting using a wall-mounted stadiometer (SECA, Ontario, CA). Next, a fasting capillary blood sample (~100 μl) was collected from a fingerstick using Safe-T-Fill® Lithium Heparin Mini Capillary Collection centrifuge tubes (RAM Scientific, Yonkers, NY). After fasting blood sample collection, participants consumed one of the four bean and white rice test meal options within 5–10 minutes under researcher supervision. Whole blood glucose concentrations were analyzed at baseline and at 30, 60, 90, 120, 150, and 180 minutes post-treatment using a Yellow Springs Instrument 2500 Stat Plus Analyzer (YSI Life Sciences, Yellow Springs, OH). All blood analyses were completed immediately after collection.

### Test meals

Participants received the four test meals in random order. Excel software was used to generate randomization sequences prior to participant recruitment by DMW (Microsoft, Redmond, WA). Three meals included one of the commercially canned *P. vulgaris* market classes: pinto beans, black beans or dark red kidney beans (Bush Brothers & Company) along with ~1/2 cup of white long grain rice (Great Value brand, Walmart). A control meal containing 180 grams or approximately 7/8 cup of steamed long grain white rice was included as the fourth meal. Long grain white rice has been found to have an average GI value of 80 ± 3 across ten studies and is considered to be a high GI food [20,26,27]. Black beans (GI value 20), pinto beans (GI value 45), and red kidney beans (GI value 20) are considered to be low GI foods [26,27]. Nutrient composition of test meals is provided in Table2. The amount of beans was standardized to provide 50 grams of available carbohydrate (CHO) while the gram weight of white rice was kept constant. Available CHO was calculated by subtracting the dietary fiber from the total CHO value listed on the manufacturer’s nutrition facts label [25,28,29]. Fifty grams of CHO is a standard amount used to test glucose response among persons with and without type 2 diabetes [19,30,31]. 

**Table 2:** Descriptive characteristics of test meals^1^

**Characteristics**	**White rice contro**l	**Pinto beans and white rice**	**Black beans and white rice**	**Kidney beans and white rice**
Total weight (g)	180.0	305.0	243.0	267.0
Rice (g)	180.0	128.0	128.0	128.0
Beans (g)	–	177.0	115.0	139.0
Energy (kcal)	232.0	273.9	257.9	277.2
Total carbohydrate (g)	49.5	59.7	55.5	58.7
Available CHO (g)	48.8	49.7	49.7	49.7
Rice (g)	48.8	34.7	34.7	34.7
Beans (g)	–	15.0	15.0	15.0
Fiber (g)	0.7	10.0	5.8	9.1
Protein (g)	4.8	11.6	9.6	10.9
Fat (g)	0.5	0.4	0.8	0.4

^1^ Nutrition information was obtained from the food’s Nutrition Facts Label; Great Value (rice) and Bush Brothers & Company (beans).

White rice was prepared in an electric automatic rice cooker based on the manufacturer’s instructions (Black & Decker RC400, Miami Lakes, FL). Rice and water amounts were standardized to gram weights for preparation consistency. Proportions of 945 g of bottled drinking water was added to 420 g of dry white rice and steamed for ~30 minutes. The canned beans (Bush Brothers & Company) were drained, but not rinsed, and heated in a microwave for 1 minute at medium power. The test meal was prepared by weighing out the cooked rice in a serving bowl, then adding the appropriate weight of warmed beans, and 15 grams of the drained can liquid for moisture. The digital scale was tared to zero to account for the serving bowl, then again after addition of each food item (Salter, Fairmont, MN).

### Statistical analysis

SPSS Statistics software version 18.0 (IBM Corporation, Somers, NY) was used for statistical analyses. The level of significance was *P* ≤ 0.05. Independent *t* tests were used to analyze data by gender and type 2 diabetes treatment type. Time point differences between fasting and post-treatment glucose concentrations were determined and incremental area under the curve (iAUC) calculations were completed using the trapezoidal rule (Table3) [32]. The iAUC for blood glucose was assessed between 0–60, 0–120, and 0–180 minutes postprandial for all participants. Multivariate analysis of variance (MANOVA) for repeated measures with time and diet as factors was used to establish differences between the four meal treatments. Effect sizes were also calculated and interpreted using Cohen’s classifications [33]. Following a significant MANOVA, paired *t* tests were used to identify differences between specific bean treatments and the rice control. All continuous variable data are reported as mean ± standard error. 

**Table 3:** Postprandial areas under the curve for blood glucose (n = 17)^1,2,3^

	**White rice control**	**Pinto beans and white rice**	**Black beans and white rice**	**Kidney beans and white rice**
0–60 min	2763.2 ± 170.0	2509.5 ± 199.0	2598.0 ± 181.8	2695.1 ± 199.0
0–120 min	6254.0 ± 448.0	5246.2 ± 409.9**	5369.2 ± 409.6*	5748.6 ± 420.1
0–180 min	7436.8 ± 622.4	5789.4 ± 488.7**	5991.6 ± 510.7**	6625.4 ± 534.6

^1^ All values are means ± standard error of the mean (SEM).

^2^ Mg * min/dL (calculated by the trapezoidal rule).

^3^ For variables in which the treatment x AUC interaction was significant, paired *t* tests were conducted to assess within-group change.

* *P* <0.05, ** *P* <0.01.

## Results

**Figure 2:**
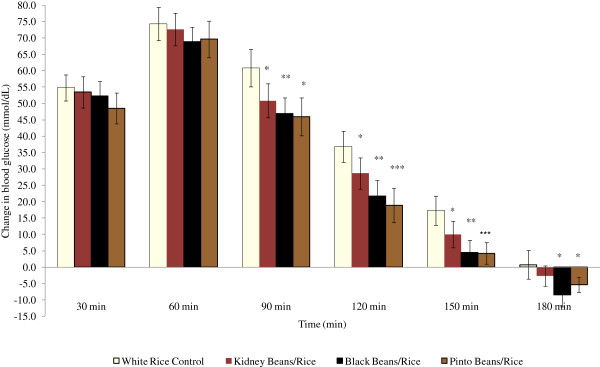
**Influence of treatments on postprandial net glucose (*n*** **= 17)**^**1**^**. **Figure Legend:  White Rice;  Kidney Beans/Rice;  Black Beans/Rice;  Pinto Beans/Rice. ^1^ All values are means ± standard error of the mean (SEM). * *P* <0.05, ** *P* <0.01, *** *P* <0.001.

Descriptive statistics at study entry for the 17 participants are shown in Table1. The majority of the participants were White (82%) and non-Hispanic (94%). Participants were also, on average, classified as obese according to their BMI values (31.8 ± 1.0 kg/m^2^). Body weight and body mass index (BMI) did not significantly differ between test days. Data were analyzed by gender and treatment type and no significant differences were seen with regard to descriptive statistics, time point glucose differences or iAUC values, so data were subsequently pooled for analysis. Time point differences in glucose concentrations were found to be significantly lower at 90 minutes postprandial for the pinto beans and rice (*P* = 0.011), black beans and rice (*P* = 0.004), and red kidney beans and rice (*P* = 0.040) as compared with the white rice control meal. Similar results were seen at 120 minutes (*P* = 0.000, 0.001 and 0.026 for the pinto beans, black beans, and red kidney beans respectively) and 150 minutes postprandial (*P* = 0.000, 0.002, and 0.0049) (Figure2). The 90 minute glucose difference had an effect size of 0.469. Medium effect sizes found at the 120 and 150 minute glucose timepoints, which were 0.634 and 0.554, respectively. The iAUC for blood glucose were assessed between 0–60, 0–120 and 0–180 minutes postprandial for all participants. Significant differences were found between the rice control meal and the pinto beans and rice and black beans and rice at 0–120 (*P =* 0.009 and 0.002) and 0–180 minutes (*P* = 0.017 and 0.007). The effect sizes (Cohen’s d) for iAUC were determined to be 0.431 for 0–120 minutes and 0.501 for 0–180 minutes.

## Discussion

Our study found bean and rice meals produce an attenuated glucose response in comparison to rice alone in equal available CHO treatments. These results reinforce those of the few existing previous studies showing intermediate responses with mixed meals of high and low GI foods. More importantly, our findings specifically demonstrate this response with traditional bean and rice combinations that are consumed widely around the world. An intermediate response is favorable to the higher response produced by white rice alone, and may help prevent the detrimental effects of prolonged glycemic elevations. Prolonged elevated glucose levels contribute to the well-known macrovascular (cardiovascular disease, peripheral vascular disease) and microvascular (nephropathy, retinopathy, neuropathy) complications associated with type 2 diabetes. Attenuating postprandial glucose changes by encouraging people with type 2 diabetes to combine traditional high GI food like rice with beans could perhaps contribute to a lower risk for the complications associated with type 2 diabetes. Also worthy of note is that all study treatments reduced the average 2 hour postprandial glucose below 140 mg/dl, which is a recommended glycemic control goal by the International Diabetes Federation [34]. This also suggests that our participants had well controlled type 2 diabetes.

*P. vulgaris* beans such as those included in this study (pinto, black and dark red kidney beans) along with white rice are a traditional food combination consumed by many in the U.S. and around the world, particularly those in Latin America and countries within the Mediterranean and Middle East. As this study demonstrates, counseling patients to exclude cultural foods like the bean and rice combination may be unwarranted for persons with type 2 diabetes. Jimenez-Cruz et al. also found that traditional Mexican foods like corn tortillas and pinto beans had a low GI, were satiating, and improved glycemic control in overweight and obese adults with type 2 diabetes [24]. Recently Mattei, Hu and Campos found that higher reporting of bean consumption in comparison to white rice was associated with reduced cardiovascular disease risk based on food frequency data from Costa Rica [35]. Retention of traditional dietary patterns that include beans may be beneficial to health, reduce type 2 diabetes complications, and improve dietary adherence [1,11,24].

An e-mail survey of Canadian dietitians found that 68% of those surveyed stated that they recommend legume consumption to individuals with diabetes, in comparison to the 87% who recommended legumes to individuals with known cardiovascular disease [36]. The American Diabetes Association recommends taking “into account personal and cultural preferences” as a goal for type 2 diabetes medical nutrition therapy [37]. However, it is not clear from the published literature whether culturally appropriate foods such as legumes are being recommended to individuals with diabetes in accordance with this goal. However, there is evidence in the public health literature of dissatisfaction or difficulty in adhering to the diabetic diet by ethnic and minority populations due to a lack of culturally appropriate recommendations with beans specifically cited as a valued part of meals and cultural identity [11-16].

Contrary to our hypothesis, the three *P. vulgaris* market classes exhibited significantly different levels of glycemic response. The pinto and black bean and rice combinations produced a lower glycemic response overall than the dark red kidney bean and rice meal despite the lower total fiber content of the black beans and the treatments being matched on available CHO content. The differences in calories, protein, and fat composition between the three bean/rice test meals are small. Woelver et al. have demonstrated that the carbohydrate content and GI of mixed meals is the central influence on glycemic response [38]. Therefore to explain differences in the observed responses variation in the specific fiber fractions of the three bean types may offer an explanation for the variation in glycemic response produced. There is some evidence that beans from the Andean center of domestication like dark red kidney may have lower levels of indigestible starch in comparison to beans with Mesoamerican origins like pinto and black. Lower levels of indigestible starch would speed up the digestion process of kidney beans in comparison to the other bean types [39]. In vitro animal evidence also indicates that red kidney beans have lesser amounts of soluble fiber and resistant starch than black beans. These components are known to slow digestion and therefore reduce postprandial glycemic response [40].

Phytochemicals and phytonutrients are associated with improvements in glycemic control [17]. These characteristics are likely to vary in the beans as well. In general, beans have high phytate levels which may bind to calcium, thus reducing it as a cofactor for α-amylase enzyme activity [30]. Inhibition of α -amylase by cooked beans has approximated that of acarbose, a popular diabetes medication [41]. Sievenpiper et al. reported in a meta-analysis that long term use of some beans normalized HbA1c almost as well as acarbose in two other meta-analysis reports [17]. Uncooked pinto beans were found to have higher levels of flavonoids than some other beans, and the sum of phenolic acids in the pinto beans was greater than chickpeas, split peas, lentils, and a variety of broad beans [42]. Data on black or red kidney beans were not reported. Pinto beans also reportedly contain high concentrations of antioxidants in comparison to chickpeas, and other non *P.vulgaris* species [43]. The observed differences in effect of the three beans highlights the importance of investigating multiple bean varieties rather than assuming all are the same. Further mechanistic work is needed on these specific varieties as well.

## Conclusion

This study demonstrates that culturally relevant *P. vulgaris* species such as pinto, dark red kidney and black beans attenuate the glycemic response to rice, a commonly consumed high GI food. As healthcare practitioners, it is vital that we are culturally competent and sensitive to the needs of others who are different from us. Cultural competency is the “ability to discover the culture of each client/patient and effectively adapt interventions to her or him” [44]. Dietary recommendations, materials and counseling should be culturally sensitive and take into account valued traditional foods such as beans, especially when the scientific evidence supports their beneficial role in the diet.

Further research should be completed regarding the physical and chemical structure of various *P. vulgaris* bean types to attempt to address the observed differences in glycemic responses. While promoting traditional foods is a non-pharmacological way to manage type 2 diabetes, knowing which beans are most effective can help improve dietary adherence with an appropriate cultural twist.

## Abbreviations

MANOVA: Multivariate analysis of variance; BMI: Body mass index; CHO: Carbohydrate; GI: Glycemic index; HbA1c: Hemoglobin A1c; iAUC: incremental area under the curve.

## Competing interests

The authors declare that they have no competing interests.

## Author’s contributions

DMW, AMH, SVT designed the research study. SVT conducted the majority of the data collection with assistance from DMW. SVT was responsible for data entry and preliminary analysis. SVT, DMW and AMH wrote the paper. DMW had primary responsibility for final content. All authors read and approved the final manuscript.
